# Evaluating SCUBE-1 as Predictive Biomarker for Hypertension-Mediated Organ Damage: A Comparative Study

**DOI:** 10.31083/RCM25832

**Published:** 2025-01-07

**Authors:** Betül Ayça Yamak, Mustafa Candemir, Emrullah Kızıltunç, Hüseyin Baran Özdemir, Özlem Gülbahar, Asife Şahinarslan

**Affiliations:** ^1^Department of Cardiology, Gazi University Faculty of Medicine, 06560 Ankara, Turkey; ^2^Department of Ophthalmology, Gazi University Faculty of Medicine, 06560 Ankara, Turkey; ^3^Department of Biochemistry, Gazi University Faculty of Medicine, 06560 Ankara, Turkey

**Keywords:** biomarker, endothelial dysfunction, hypertension, hypertension-mediated organ damage, SCUBE-1

## Abstract

**Background:**

Hypertension-mediated organ damage (HMOD) is a critical complication of hypertension that can present with cardiac, retinal, and renal manifestations and affect patient outcomes. Serum signal peptide, CUB (complement C1r/C1s, Uegf, and Bmp1) domain, and epidermal growth factor-like domain-containing protein 1 (SCUBE-1), a novel biomarker implicated in vascular pathology, shows promise for detecting HMOD. This study aims to explore the relation between SCUBE-1 levels and HMOD in hypertensive patients.

**Methods:**

This cross-sectional study included 115 participants, comprising 79 hypertensive patients and 36 healthy controls. The hypertensive patients were divided into two groups based on HMOD presence. SCUBE-1 levels were measured to evaluate their diagnostic utility in detecting HMOD.

**Results:**

Hypertensive patients exhibited significantly higher SCUBE-1 levels than controls (160.70 ng/mL vs. 75.64 ng/mL, *p* < 0.001). Among these patients, those with HMOD (cardiac, retinal, and renal) displayed even higher SCUBE-1 levels (311.27 ng/mL, range 137.86–460 ng/mL) compared to those without HMOD (142.53 ng/mL, range 110.56–178.19 ng/mL). Receiver operating characteristic curve analysis indicated that SCUBE-1 levels have significant diagnostic potential for differentiating between hypertensive patients with and without HMOD with area under the curve values of 0.722 for cardiac, 0.761 for retinal, and 0.707 for renal damage.

**Conclusions:**

Our study has revealed that SCUBE-1 levels are significantly elevated in hypertensive patients, particularly those with HMOD. The findings support the potential of SCUBE-1 as a valuable biomarker for predicting organ damage in hypertensive patients.

## 1. Introduction

Hypertension (HT) is one of the most common causes of morbidity and mortality worldwide [1, 2]. HT is associated with cardiovascular events including ischemic heart disease, stroke, heart failure, and abdominal aortic aneurysm. The most common causes of HT-related deaths are ischemic heart disease, hemorrhagic stroke, and ischemic stroke [2]. Additionally, high systolic blood pressure (SBP) plays a significant role in global disability, resulting in 218 million disability-adjusted life years for both sexes [3].

Untreated high blood pressure can cause organ damage including the heart, brain, eyes, kidneys, and arteries [4]. HT-mediated organ damage (HMOD) also increases the risk of cardiovascular disease [5]. If blood pressure is left uncontrolled, HMOD can result in irreversible damage. However, controlling blood pressure through medication and lifestyle changes can prevent further damage and reduce the risk of cardiovascular disease [4, 6].

Recent studies have highlighted the role of the inflammatory induced signal peptide, CUB (complement C1r/C1s, Uegf, and Bmp1) domain, and epidermal growth factor like domain-containing protein 1 (SCUBE-1) in the pathophysiology of HT [7, 8]. Specifically, SCUBE-1 was identified in a project assessing gene expression in human endothelial cells; this protein is characterized by 10 epidermal growth factor-like domains and a CUB domain at the carboxyl terminus [7]. Vascular endothelial cells play an essential in various pathophysiological processes such as angiogenesis, inflammation, and vascular diseases. Predominantly expressed in endothelial cells, SCUBE-1 serves as a marker for inflammation and endothelial dysfunction [9]. It has been shown that SCUBE-1 levels increase in response to inflammation or stress in these cells. It has been shown that SCUBE-1 levels elevate in response to inflammation or stress in these cells. Furthermore, SCUBE-1 is critical for the body’s reaction to chronic microvascular trauma and inflammation [7, 8, 10]. The significance of SCUBE-1 as a marker of endothelial dysfunction and inflammation is also supported by its association with elevated levels in conditions such as ischemic heart disease and HT [8, 9].

Studies have demonstrated that endothelial dysfunction is prevalent in individuals with HT [11, 12]. Additionally, HT is known to predispose individuals to endothelial dysfunction, which is implicated in HMOD pathophysiology [12]. Given this context, this study aims to investigate the potential relationship between SCUBE-1 levels and HMOD.

## 2. Methods

### 2.1 Study Design and Population

The study was designed as a cross-sectional analysis of the association between SCUBE-1 levels and HMOD. A total of 115 patients who were admitted to Gazi University cardiology outpatient clinic between February 2022 to February 2023 and met the inclusion criteria were included in the study. Ambulatory blood pressure monitoring (ABPM) was performed on patients in addition to evaluation for end organ damage. Subsequently, SCUBE-1 levels were measured in all patients.

The study enrolled patients who were either previously diagnosed with HT or newly diagnosed upon admission, following the European Society of Cardiology guidelines [13]. Hypertensive patients were stratified into two groups based on the presence or absence of HMOD. The control group comprised individuals visiting the outpatient clinic for various complaints without comorbidities and were confirmed to not have HT diagnoses based on ABPM. Patients with conditions that could confound the study results were excluded. These conditions included acute coronary syndrome, acute heart failure, congenital heart disease, severe heart valve disease, atrial fibrillation, diabetes mellitus, morbid obesity, asthma or chronic obstructive pulmonary disease, psychiatric diseases, neurological diseases, endocrinological diseases, alcohol or substance addiction, acute infection and connective tissue diseases.

### 2.2 Data Collection

#### 2.2.1 Laboratory Assessments

Laboratory evaluations of the patients included complete blood count, kidney and liver function tests, fasting blood sugar levels, lipid profiles, and albumin: creatinine ratio (ACR) from spot urine samples. Three milliliters (mL) of venous blood were drawn into citrate tubes for SCUBE-1 from both patients and healthy control subjects. Blood samples were centrifuged at +4 °C for 20 minutes using Thermo Scientific SL40R centrifuge (Thermo Fisher Scientific, Waltham, MA, USA). After centrifugation, plasma samples were transferred to Eppendorf tubes and stored at –80 °C until SCUBE1 testing was performed. SCUBE1 levels were determined using a specific enzyme-linked immunosorbent assay (ELISA) kit with a detection range of 1 ng/mL to 400 ng/mL (E3142Hu, BT-LAB, Shanghai, China). The ELISA assay was performed according to the manufacturer’s instructions. Briefly, plasma samples and standards were added to the appropriate wells of the ELISA plate. After an incubation period, unbound substances were washed away. A SCUBE1-specific enzyme-linked antibody was added, followed by a substrate solution. The absorbance of samples was measured at 450 nm using a VERSAmax tunable microplate reader (Molecular Devices, Sunnyvale, CA, USA). Results obtained from the assay were expressed in nanograms per milliliter (ng/mL).

#### 2.2.2 Ambulatory Blood Pressure Monitoring

Blood pressure monitoring was performed non-invasively using a validated device (GE Healthcare Tonoport V, Berlin, Germany). The monitoring process involved attaching the device to patients between 08:00 and 10:00 and removing it after 24 hours.

The device was set up to measure blood pressure every 20 minutes throughout the day, from 06:00 to 21:59, and every 30 minutes at night, from 22:00 to 05:59.

The minimum requirements for ABPM recordings were established as follows:

(1) At least 70% of measurements should be valid during the 24-hour period.

(2) There should be at least 20 valid measurements, with at least 2 per hour while awake.

(3) There should be at least 7 valid measurements, with at least one per hour while asleep.

Diagnostic thresholds for HT based on ABPM are daytime SBP/diastolic blood pressure (DBP) ≥135/85 mmHg, nighttime SBP/DBP ≥120/70 mmHg, and 24-hour mean SBP/DBP ≥130/80 mmHg [13].

### 2.3 Diagnostic Criteria for HMOD

Hypertensive patients underwent an assessment for end-organ (cardiac, retinal, and renal) damage. Subsequently, the hypertensive patients were divided into two groups based on the presence or absence of HMOD.

#### 2.3.1 Hypertensive Cardiomyopathy

All patients participating in the study underwent two-dimensional, M-mode, and tissue Doppler echocardiographic assessments performed by the same cardiologist, utilizing a Vivid 7 Digital ultrasound device (GE Healthcare, Horten, Norway) with a 3.5 MHz S5-1 transducer. Images were recorded over three cycles.

The following echocardiographic parameters were obtained:

Measurements of left ventricle (LV) dimensions, interventricular septum thickness (IVST), and LV posterior wall thickness (PWT) were obtained through vertical sections along the long axis from the mitral leaf tips using M-mode. LV mass and LV mass indexes (LVMI) were noted. Values for LVMI >115 g/m^2^ in men and >95 g/m^2^ in women indicated left ventricular hypertrophy (LVH) [13]. Relative wall thickness (RWT) was calculated for geometry analysis using the formula RWT = 2 × PWT diameter/LV end-diastolic diameter [14]. Based on their LV geometry, patients were classified as normal, concentric remodeling, concentric hypertrophy, or eccentric hypertrophy [14].

Mitral flow velocities were recorded with pulsed-wave Doppler by placing the sample 1 cm above the mitral annular line between the mitral valve tips. E and A wave velocities, deceleration time, and isovolumic relaxation time were recorded. Mean E’ was calculated using tissue Doppler for the septal and lateral mitral annulus. Tricuspid relaxation velocity and systolic pulmonary artery pressure values were measured. Left atrial volume was recorded by drawing endocardial boundaries from apical four- and two-chamber windows. The left atrial volume index was calculated by dividing by body surface area. Diastolic dysfunction was graded according to the recommendations of the American Society of Echocardiography and the European Association of Cardiovascular Imaging for Evaluation of Left Ventricular Diastolic Function by Echocardiography [15]. Patients with LVH or diastolic dysfunction were considered to have hypertensive cardiomyopathy.

#### 2.3.2 Hypertensive Retinopathy

Patients with HT underwent evaluations for hypertensive retinopathy by fundoscopy performed by the same ophthalmologist. Patients were evaluated for arterial narrowing, venous dilation, retinal hemorrhages, exudates, cotton wool spots, optic disc changes, macular edema, retinal detachment, and neovascularization.

#### 2.3.3 Hypertensive Nephropathy

Renal function parameters, including glomerular filtration rate (GFR), creatinine, and spot urine ACR, were recorded to assess hypertensive nephropathy. The diagnosis of HT-induced renal injury was based on the presence of albuminuria or reduced renal function (GFR of less than 60 mL/min/1.73 m^2^) and the exclusion of primary renal disease. ACR was measured from a spot urine sample (preferably early morning urine) to measure urinary albumin excretion. An ACR of less than 30 mg/g was considered normal. A ratio between 30 and 300 mg/g indicated microalbuminuria, an early sign of hypertensive nephropathy. Values above 300 mg/g were classified as macroalbuminuria, signifying more advanced kidney damage [16].

### 2.4 Statistical Analysis

All data were analyzed using SPSS version 25.0 (IBM, Armonk, NY, USA). Continuous variables were presented as mean ± standard deviation or medians (interquartile range) according to the distribution pattern, and categorical data were expressed as percentages or frequencies. Continuous variables were examined using the Kolmogorov–Smirnov test to confirm normal distribution. Clinical features and laboratory measurements were compared between groups using the Student’s *t*-test, Mann-Whitney U test, or the chi-square test as appropriate. Multivariable logistic regression analyses were performed to determine the independent predictors of HMOD in patients with HT. The capacity of SCUBE-1 value in predicting the presence of HMOD was analyzed using receiver operating characteristic (ROC) curve analysis. A two-tailed *p*-value of ≤0.05 indicates that the related parameter is statistically significant.

## 3. Results

### 3.1 Analysis of Participants with HT Compared to the Control Group

Our study analyzed a cohort of 115 participants, divided into two groups: those with HT and controls. Demographics, clinical characteristics, and laboratory values are shown in Table 1. The control group included 36 participants with a mean age of 48.2 ± 8.8 years, while the HT group comprised 79 participants with a mean age of 49.9 ± 11.7 years. Regarding sex distribution, males constituted 33 % of the control group and 48 % of the HT group.

**Table 1.** Demographics, clinical characteristics, and laboratory values of study participants*.

Variable	Control group (n = 36)	HT group (n = 79)	*p*-value
Age, years	48.2 ± 8.8	49.9 ± 11.7	0.434
Sex (male), n (%)	12 (33.3)	38 (48.1)	0.138
BMI, (kg/m^2^)	27.77 ± 4.59	28.91 ± 4.63	0.223
Smoker, n (%)	12 (33.3)	27 (34.2)	0.929
Hemoglobin, g/dL	13.73 ± 1.73	14.37 ± 1.55	0.061
Platelet, ×10^3^/mm^3^	263.13 ± 67.18	267.89 ± 62.28	0.712
WBC, ×10^3^/mm^3^	7.60 ± 1.97	7.67 ± 2.03	0.859
Fasting blood glucose, mg/dL	89 (81–106)	94 (84–106)	0.558
HbA1c, %	5.7 (5.4–5.9)	5.8 (5.4–6.1)	0.286
BUN, mg/dL	13 (10.25–15)	14 (12–17)	0.176
Creatinine, mg/dL	0.73 ± 0.16	0.77 ± 0.15	0.151
GFR, mL/min/1.73 m^2^	87.94 ± 5.16	87.58 ± 5.38	0.737
Uric acid, mg/dL	4.75 (3.60–5.90)	5.40 (4.40–6.50)	0.005
Sodium, mmol/L	140.22 ± 1.92	140.23 ± 1.80	0.985
Potassium, mmol/L	4.45 (4.13–4.70)	4.30 (4.10–4.54)	0.253
AST, IU/L	21.50 (17–26)	21 (17–24)	0.597
ALT, IU/L	20 (16–28)	21 (17–29)	0.623
Total protein, g/dL	7.32 ± 0.41	7.32 ± 0.42	0.851
Albumin, g/dL	4.49 ± 0.24	4.45 ± 0.25	0.422
Calcium, mg/dL	9.48 ± 0.40	9.67 ± 1.26	0.228
TSH, mIU/L	1.64 (1.29–2.82)	1.80 (1.13–2.72)	0.995
Total cholesterol, mg/dL	180 (166.25–216.75)	197 (178–237)	0.070
Triglycerides, mg/dL	124 (85–177.75)	143 (105–214)	0.150
LDL, mg/dL	109.50 (93–129.25)	123 (102–143)	0.083
HDL, mg/dL	49.90 (40.12–57.37)	48 (38.90–55.70)	0.660
Urine microalbumin, mg/dL	0.63 (0.50–2.61)	1.64 (0.50–14)	0.010
Urine protein, mg/dL	9 (4.25–14.93)	8.50 (6.50–14.10)	0.779
Urine creatinine, mg/dL	103.58 (43.94–152.93)	102.15 (67.06–150)	0.496
Urine protein/creatinine ratio, mg/g	0.11 (0.07–0.14)	0.09 (0.06–0.13)	0.259
Urine albumin/creatinine ratio, mg/g	7.55 (2.42–18.85)	12.37 (7.64–21.66)	0.027
SCUBE-1 levels, ng/mL	75.64 (52.71–110.36)	160.70 (124.12–319.80)	<0.001

* Results expressed as mean ± standard deviation, median (interquartile range), or frequency (%). ALT, alanine aminotransferase; AST, aspartate transaminase; BMI, body mass index; BUN, blood urea nitrogen; GFR, glomerular filtration rate; HbA1c, hemoglobin A1c; HDL, high-density lipoprotein; HT, hypertension; LDL, low-density lipoprotein; SCUBE-1, signal peptide, CUB (complement C1r/C1s, Uegf, and Bmp1) domain, and epidermal growth factor like domain-containing protein 1; TSH, thyroid stimulating hormone; WBC, white blood cell; IU, international unit.

While most laboratory parameters did not show significant differences between the two groups, there were two notable exceptions. Urinary microalbumin levels were significantly elevated in the HT group, with levels of 1.64 mg/dL (range 0.50–14 mg/dL) versus 0.63 mg/dL (range 0.50–2.61 mg/dL), respectively (*p* = 0.010). Similarly, SCUBE-1 levels were significantly elevated in the HT group, with a mean of 160.70 ng/mL (range 124.12–319.80 ng/mL) compared to 75.64 ng/mL (range 52.71–110.36 ng/ml) in the control group (*p* < 0.001).

The study revealed the presence of HMOD in 49% of patients diagnosed with HT. Specific organ changes were as follows: cardiac changes were identified in 24 patients, retinal changes in 8, and renal changes in 12, as detailed in Table 2. Among these patients, two exhibited both cardiac and renal changes, three had both cardiac and retinal changes, and one had both renal and retinal changes.

**Table 2.** **Organ damage catgegories in patients with HT**.

Organ damage type	Number of patients with HMOD	Percentage of total HMOD cases (%)
Only heart	19	48.7
Only eyes	4	10
Only kidney	10	25.6
Heart + Eyes	3	7.7
Heart + Kidney	2	5.1
Kidney + Eyes	1	2.6
Heart + Eyes + Kidney	0	0
Total HMOD cases	39	100

HMOD, hypertension-mediated organ damage; HT, hypertension.

No statistically significant differences were observed between the control and HT groups in aortic diameter, ascending aorta diameter, LV end-diastolic diameter, end-systolic diameter, and left atrial diameter. Similarly, no significant differences were observed in left ventricular ejection fraction between the groups. However, indices for IVST, PWT, RWT, and LVMI were significantly higher in the HT group. Among diastolic function parameters, the E/A ratio and deceleration time did not differ significantly between the groups.

Notably, septal e’ and lateral e’ velocities were higher in the control group, while tricuspid regurgitant velocity and E/e’ ratio was significantly higher in the HT group (Table 3).

**Table 3.** Transthoracic echocardiographic parameters in patients with HT*.

Parameter	Control group (n = 36)	HT group (n = 79)	*p*-value
Aorta, cm	27.86 ± 2.97	28.27 ± 2.67	0.463
Ascending aorta, cm	34.53 ± 3.34	34.42 ± 3.35	0.937
LVED, cm	4.65 (4.20–4.87)	4.50 (4.30–4.70)	0.398
LVES, cm	2.69 ± 0.47	2.82 ± 0.42	0.103
IVST, cm	1 (0.90–1.17)	1.10 (1–1.20)	0.019
PWT, cm	0.90 (0.80–1.07)	1 (0.9–1.10)	0.019
RWT	0.42 ± 0.08	0.46 ± 0.08	0.014
LVEF, %	66.19 ± 4.78	64.48 ± 4.88	0.081
LA, cm	3.62 ± 0.41	3.72 ± 0.41	0.216
LA volume, mL	43.75 (37.05–63.04)	52.01 (45.29–66.39)	<0.001
LAVI, mL/m^2^	23.84 (20.12–31.34)	26.54 (21.95–31.42)	0.579
E/A ratio	0.90 (0.80–1.33)	0.99 (0.76–1.30)	0.531
DT, msec	205 (175–232.25)	205 (190–250)	0.295
TRV, m/sec	1.95 ± 0.42	2.22 ± 0.60	0.007
Septal e’, cm/s	7.89 ± 1.81	6.88 ± 2.04	0.012
Lateral e’, cm/s	11.80 ± 3.08	10.41 ± 3.05	0.026
E/e’ ratio	7.20 (6–8.75)	8 (7–12)	0.019
LVM, g	142.89 (118.67–190.54)	163.98 (132.82–193.25)	0.195
LVMI, g/m^2^	76.70 (62.27–102)	88 (72–105.86)	0.039

* Results are expressed as mean ± standard deviation, median (interquartile range), or frequency (%). HT, hypertension; LVED, left ventricular end-diastolic diameter; LVES, left ventricular end-systolic diameter; IVST, interventricular septum thickness; PWT, posterior wall thickness; RWT, relative wall thickness; LVEF, left ventricular ejection fraction; LA, left atrium; LAVI, left atrium volume index; DT, deceleration time; LVM, left ventricular mass; LVMI, left ventricular mass index; TRV, tricuspid regurgitation velocity.

### 3.2 Analysis of Participants with HT and HMOD Compared to HT Participants without HMOD

Among the 79 patients with HT examined, 39 showed detectable organ damage, comprising the HMOD group. The urinary ACR was notably higher in the HMOD group (19.17 mg/g vs. 9.04 mg/g). Additionally, SCUBE-1 levels were significantly higher in the HMOD group at 311.27 ng/mL (range 137.86–460 ng/mL) compared to 142.53 ng/mL for the no-HMOD group (range 110.56–178.19 ng/mL) (Table 4). Patients with both HMOD and HT exhibited significantly higher measurements of IVST, PWT, RWT, left atrial volume, tricuspid regurgitation velocity, E/e’ ratio, and LVMI, with lower measurements for septal e’ and lateral e’ velocity compared to those without HMOD (Table 5). In addition, multivariable logistic regression analysis confirmed that SCUBE-1 was an independent predictor of HMOD (Table 6).

**Table 4.** Demographics, clinical characteristics, and laboratory measurements in patients with HT*.

Variable	HT with HMOD (n = 39)	HT without HMOD (n = 40)	*p* value
Age, years	50.1 ± 12.3	49.7 ± 11.3	0.881
Sex (male), n (%)	22 (56.4)	16 (40)	0.144
BMI, (kg/m^2^)	27.75 ± 4.77	30.04 ± 4.24	0.027
Smoking, n (%)	14 (35.9)	13 (32.5)	0.750
Hemoglobin, g/dL	14.57 ± 1.48	14.17 ± 1.6	0.247
Platelet count, ×10^3^/mm^3^	262.12 ± 5.94	273.52 ± 65.16	0.277
WBC count, ×10^3^/mm^3^	7.72 ± 2.11	7.6 ± 1.9	0.836
Fasting blood sugar, mg/dL	94 (84–103)	93 (84–106)	0.746
HbA1c, %	5.8 (5.4–6.1)	5.7 (5.4–6.2)	0.798
BUN, mg/dL	15.17 ± 4.47	13.93 ± 3.43	0.168
Creatinine, mg/dL	0.79 ± 0.15	0.75 ± 0.14	0.230
GFR, mL/min/1.73 m^2^	87.56 ± 5.83	87.60 ± 4.98	0.977
Uric acid, mg/dL	5.60 (4.60–7)	5.20 (4.30–6.35)	0.359
Sodium, mmol/L	140.18 ± 1.73	140.28 ± 1.89	0.816
Potassium, mmol/L	4.32 ± 0.38	4.35 ± 0.34	0.778
AST, IU/L	21.77 ± 6.57	21.20 ± 4.78	0.661
ALT, IU/L	21 (15–28)	21 (18–29.75)	0.467
Total protein, g/dL	7.36 ± 0.40	7.29 ± 0.42	0.450
Albumin, g/dL	4.44 ± 0.26	4.47 ± 0.24	0.552
Calcium, mg/dL	9.91 ± 1.74	9.54 ± 0.46	0.206
TSH, mIU/L	1.75 (1.29–2.80)	1.86 (1.04–2.57)	0.666
Total cholesterol, mg/dL	192 (177–222)	209 (178.25–250.05)	0.312
Triglyceride, mg/dL	143 (115–238)	141.50 (98.75–212.25)	0.433
LDL, mg/dL	119 (98–136)	127.50 (109.50–153.50)	0.239
HDL, mg/dL	47.39 ± 12.41	50.06 ± 13.98	0.373
Urine microalbumin, mg/dL	12.29 (1.36–81.08)	0.95 (0.50–1.69)	<0.001
Urine protein, mg/dL	10.77 (6.90–22)	7.49 (6.18–11.57)	0.038
Urine creatinine, mg/dL	100.40 (67.21–158.26)	107.16 (60.65–146.33)	0.837
Urine protein/creatinine ratio, mg/g	0.10 (0.06–0.18)	0.07 (0.05–0.11)	0.012
Urine albumin/creatinine ratio, mg/g	19.17 (10.87–43.07)	9.04 (4.80–13.12)	<0.001
SCUBE-1 levels, ng/mL	311.27 (137.86–460)	142.53 (110.56–178.19)	<0.001

* Results expressed as mean ± standard deviation, median (interquartile range), or frequency (%). HT, hypertension; HMOD, hypertension-mediated organ damage; BMI, body mass index; WBC, white blood cell; HbA1c, hemoglobin A1c; BUN, blood urea nitrogen; GFR, glomerular filtration rate; ALT, alanine aminotransferase; AST, aspartate transaminase; TSH, thyroid stimulating hormone; HDL, high-density lipoprotein; LDL, low-density lipoprotein; SCUBE-1, signal peptide, CUB (complement C1r/C1s, Uegf, and Bmp1) domain, and epidermal growth factor like domain-containing protein 1.

**Table 5.** Transthoracic echocardiographic parameters in patients with HT, with and without HMOD*.

Echocardiographic parameter	HT with HMOD (n = 39)	HT without HMOD (n = 40)	*p* value
Aorta, cm	28.38 ± 2.94	28.15 ± 2.42	0.700
Ascending aorta, cm	34.41 ± 3.41	34.43 ± 3.32	0.985
LVED, cm	4.54 ± 0.41	4.52 ± 0.40	0.862
LVES, cm	2.76 ± 0.43	2.87 ± 0.41	0.241
IVST, cm	1.20 (1.10–1.30)	1.05 (1–1.10)	<0.001
PWT, cm	1.10 (1–1.20)	1 (0.9–1.06)	0.001
RWT	0.49 ± 0.10	0.42 ± 0.04	<0.001
LVEF, %	64.26 ± 4.67	64.70 ± 5.12	0.689
LA, cm	3.67 ± 0.43	3.77 ± 0.40	0.314
LA volume, mL	58.09 ± 15.36	51.75 ± 15.85	0.075
LAVI, mL/m^2^	26.96 (20.90–31.42)	26.27 (22.02–31.29)	0.984
E/A ratio	0.9 (0.8–1.30)	0.99 (0.73–1.25)	0.902
DT, msec	225 (190–257)	202 (183.50–229)	0.255
TRV, m/sn	2.49 ± 0.60	1.95 ± 0.45	<0.001
Septal e’, cm/s	6 (5–8)	7.50 (6–9)	0.012
Lateral e’, cm/s	9 (7–11)	11.50 (8.25–14)	0.007
E/e’ ratio	11 (8.15–16)	7 (6–8)	<0.001
LVM, g	179.96 (136.20–203.05)	155.71 (132.18–184.75)	0.077
LVMI, g/m^2^	92.34 (80–113.23)	79.73 (67.35–93.89)	0.003

* Results are expressed as mean ± standard deviation, median (interquartile range), or frequency (%). HMOD, hypertension-mediated organ damage; HT, hypertension; LVED, left ventricular end-diastolic diameter; LVES, left ventricular end-systolic diameter; IVST, interventricular septum thickness; PWT, posterior wall thickness; RWT, relative wall thickness; LVEF, left ventricular ejection fraction; LA, left atrium; LAVI, left atrium volume index; DT, deceleration time; LVM, left ventricular mass; LVMI, left ventricular mass index; TRV, tricuspid regurgitation velocity.

**Table 6.** **Logistic regression analysis of HMOD prediction factors in patients with HT**.

	Multivariable analysis			
	Odds ratio	95% Confidence interval		*p*
		Lower limit	Upper limit	
Age	1.037	0.975	1.102	0.246
Sex (male)	0.503	0.119	2.121	0.349
BMI	1.020	0.866	1.200	0.816
Smoking	2.066	0.559	7.642	0.277
HbA1c	1.004	0.346	2.914	0.995
Uric acid	1.236	0.833	1.836	0.292
LVEF	1.010	0.896	1.138	0.870
SCUBE-1 levels (ng/mL)	1.016	1.008	1.025	<0.001

HMOD, hypertension-mediated organ damage; HT, hypertension; BMI, body mass index; LVEF, left ventricle ejection fraction; HbA1c, hemoglobin A1c; SCUBE-1, signal peptide, CUB (complement C1r/C1s, Uegf, and Bmp1) domain, and epidermal growth factor like domain-containing protein 1.

**Fig. 1.**
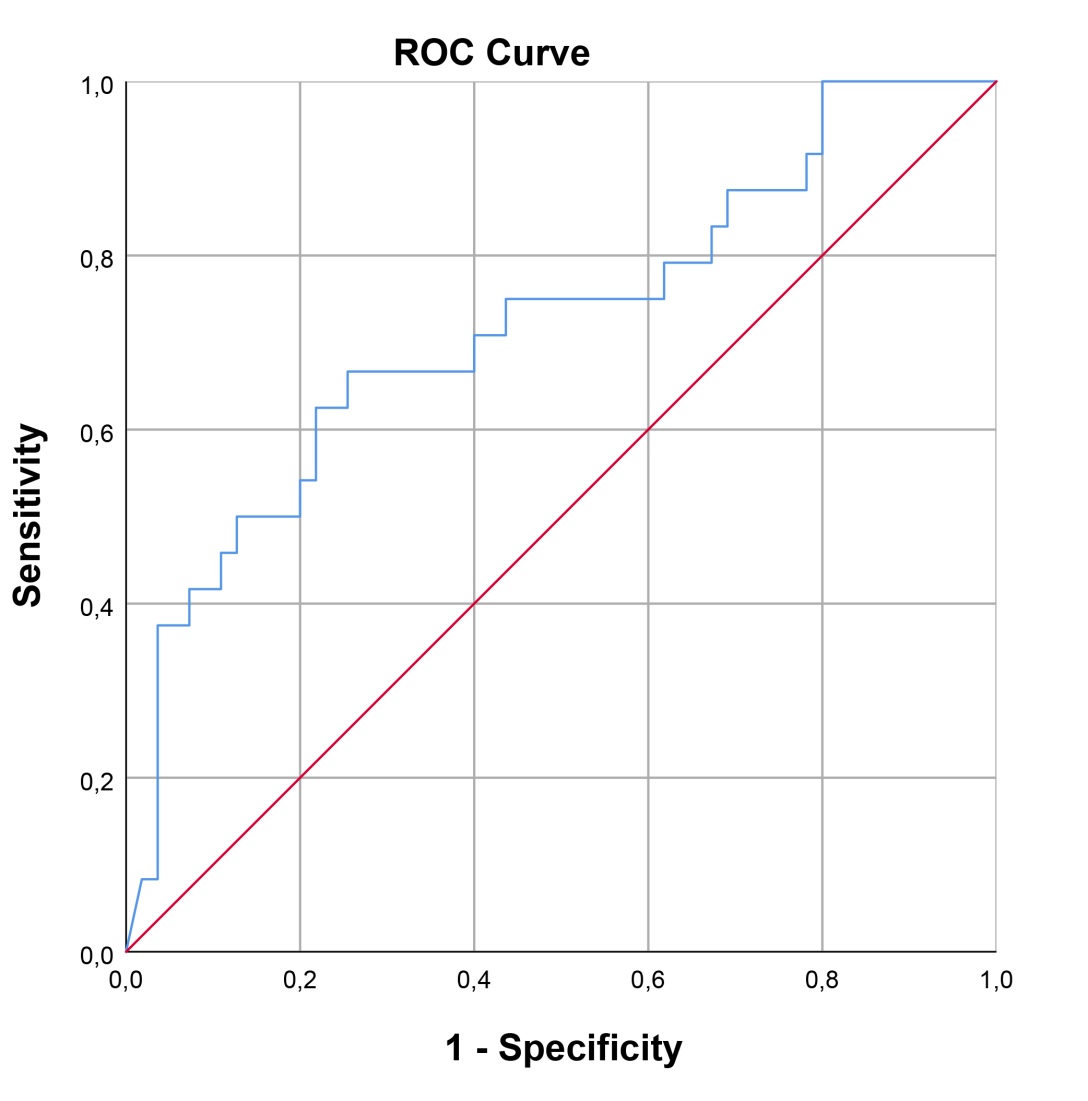
**ROC curve analysis for SCUBE-1 as a biomarker of cardiac damage in HT**. This figure displays the sensitivity as a function of specificity for SCUBE-1 levels as a biomarker. The AUC was found to be 0.722 (95% CI, 0.593–0.851; *p* = 0.002). ROC, receiver operating characteristic; AUC, area under the curve; HT, hypertension; SCUBE-1, signal peptide, CUB (complement C1r/C1s, Uegf, and Bmp1) domain, and epidermal growth factor like domain-containing protein 1.

**Fig. 2.**
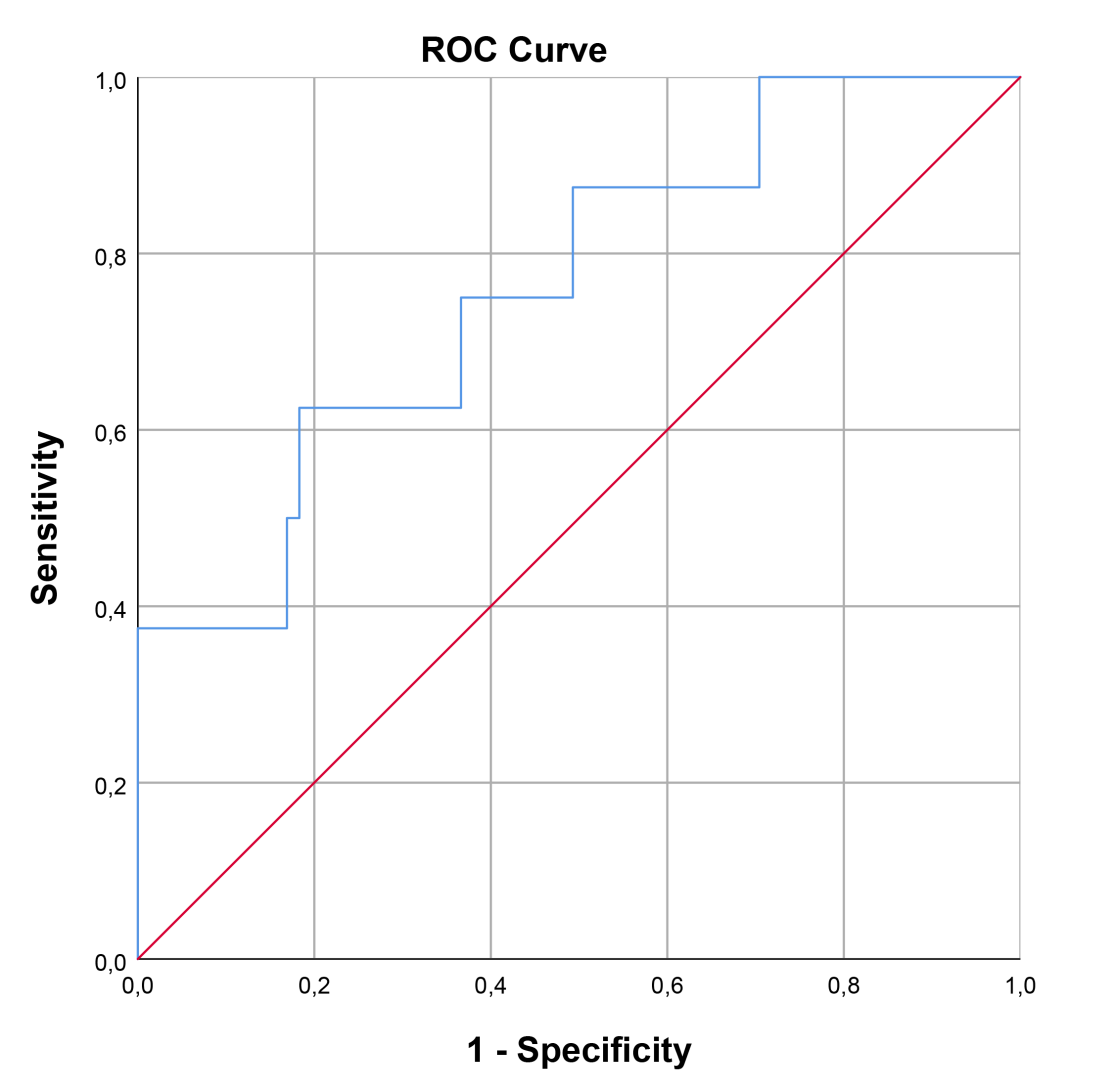
**ROC curve analysis for SCUBE-1 as a biomarker for retinal damage in HT**. This figure displays the sensitivity as a function of specificity for SCUBE-1 levels as a biomarker. The AUC was found to be 0.761 (95% CI, 0.582–0.939; *p* = 0.016). ROC, receiver operating characteristic; AUC, area under the curve; HT, hypertension; SCUBE-1, signal peptide, CUB (complement C1r/C1s, Uegf, and Bmp1) domain, and epidermal growth factor like domain-containing protein 1.

**Fig. 3.**
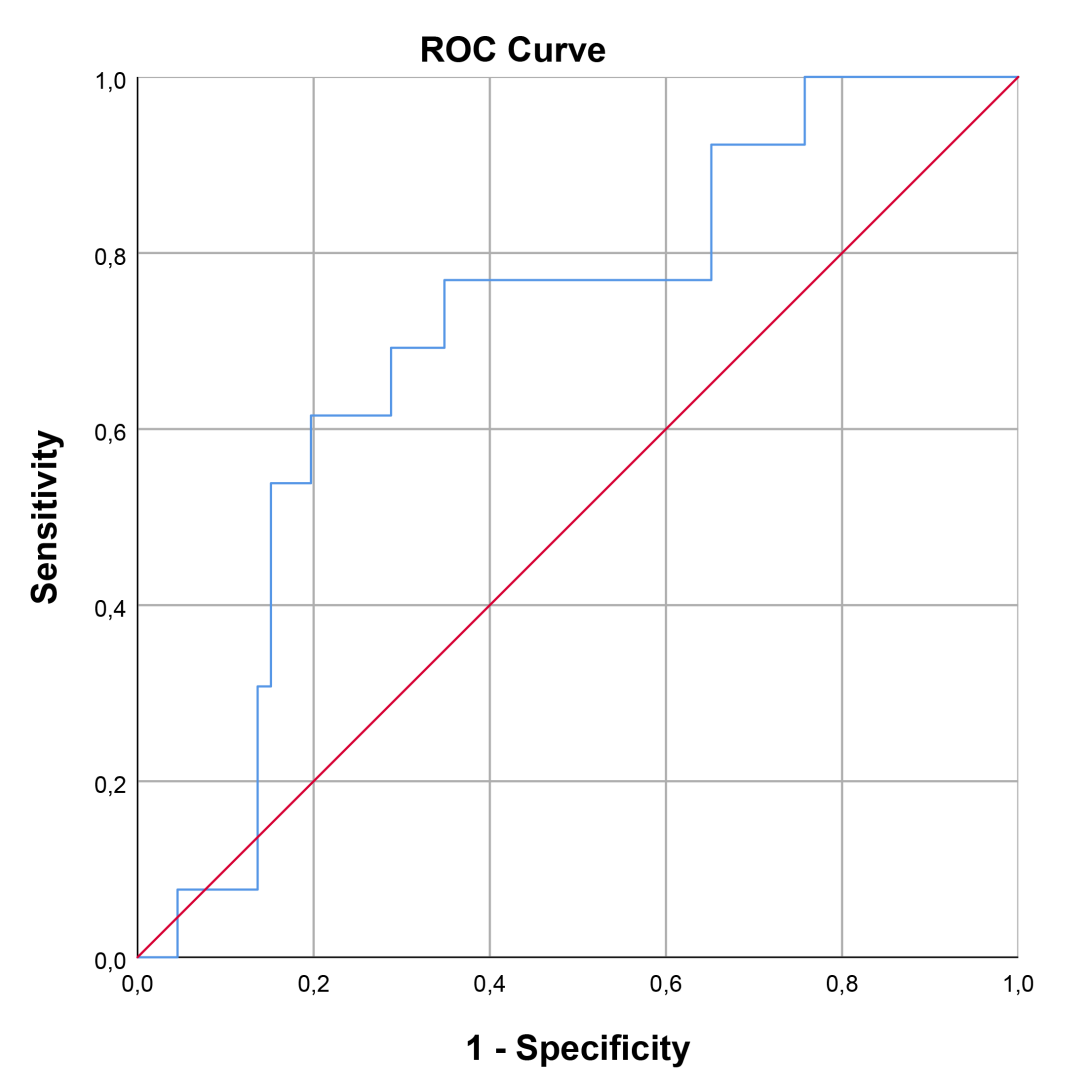
**ROC curve analysis for SCUBE-1 as a biomarker for renal damage in HT**. This figure displays the sensitivity as a function of specificity for SCUBE-1 levels as a biomarker. The AUC was found to be 0.707 (95% CI, 0.563–0.852; *p* = 0.019). ROC, receiver operating characteristic; AUC, area under the curve; HT, hypertension; SCUBE-1, signal peptide, CUB (complement C1r/C1s, Uegf, and Bmp1) domain, and epidermal growth factor like domain-containing protein 1.

The optimal cut-off value for SCUBE-1 levels in diagnosing cardiac damage was determined to be 229.74 ng/mL, exhibiting a sensitivity of 66.7% and a specificity of 74.5%. For retinal damage, the optimal cut-off value was higher, at 362.35 ng/mL, with a sensitivity of 62.5% and a specificity of 81.7%. In the case of renal damage, the cut-off value for renal damage was set at 191.19 ng/mL, achieving a sensitivity of 76.9% and a specificity of 65.2%. The area under the curve (AUC) values indicating diagnostic performance were 0.722 (95% confidence interval [CI], 0.593–0.851; *p* = 0.002) for cardiac damage, 0.761 (95% CI, 0.582–0.939; *p* = 0.016) for retinal damage, and 0.707 (95% CI, 0.563–0.852; *p* = 0.019) for renal damage. These findings, including the diagnostic cut-off values and AUCs for different types of damage, are detailed in Table 7 and illustrated through ROC curve analysis in Figs. 1,2,3.

**Table 7.** **Diagnostic performance of SCUBE-1 levels for different HMOD types based on ROC curve analysis**.

	Cut-off value (ng/mL)	Sensitivity	Specificity	AUC	95% Confidence interval		*p*
					Lower limit	Upper limit	
Cardiac	229.74	66.7	74.5	0.722	0.593	0.851	0.002
Retinal	362.35	62.5	81.7	0.761	0.582	0.939	0.016
Renal	191.19	76.9	65.2	0.707	0.563	0.852	0.019

HMOD, hypertension-mediated organ damage; ROC, receiver operating characteristic; AUC, area under the curve; SCUBE-1, signal peptide, CUB (complement C1r/C1s, Uegf, and Bmp1) domain, and epidermal growth factor like domain-containing protein 1.

## 4. Discussion

This study demonstrated that patients with HT who have HMOD exhibited higher SCUBE-1 levels compared to those without HMOD. Additionally, SCUBE-1 levels were elevated in HT patients relative to the healthy control group. Importantly, SCUBE-1 was identified as an independent predictor of HMOD.

Despite extensive research, the complex multifactorial pathophysiology of HT remains unclear. Recently, there has been increasing focus on endothelial dysfunction, which is thought to play a critical role in both the development of HT and its subsequent complications. Endothelial dysfunction includes not only disrupted endothelium-dependent vasodilation, but also activation of chronic low-grade inflammation [17]. This type of inflammation contributes to the development of HMOD through vascular remodeling [18, 19]. Additionally, the relationship between blood pressure levels and platelet activation has been established in previous studies. There is evidence that intracellular signaling abnormalities in platelets from patients with HT, primarily associated with high shear stress and endothelial dysfunction, contribute to these processes. Platelet activation is also implicated in HT-mediated angiogenesis and organ damage [20].

SCUBE-1 is a cell-surface glycoprotein expressed primarily in platelets and endothelial cells, and plays an essential role in both vascular biology and endothelial function [7]. In Dai *et al*.’s study [21], SCUBE-1 levels were found to be elevated in patients with acute coronary syndrome and stroke, but not chronic coronary artery disease. This result was attributed to the severe inflammation and platelet activation observed under acute conditions. Thus, SCUBE-1 is a promising novel biomarker for assessing inflammation and platelet activation.

SCUBE-1 levels are significantly higher in newly diagnosed patients with primary HT when compared to individuals with normal blood pressure levels, suggesting a potential role of SCUBE-1 in the pathogenesis of HT [8]. Additionally, elevated SCUBE-1 levels have been observed in patients with non-dipper blood pressure patterns. Although it is known that non-dipper hypertensive patients experience worse outcomes, HMOD was not assessed in those studies [22, 23]. Our results support these findings, reinforcing the association between SCUBE-1 and HT. However, we expand on this understanding by linking SCUBE-1 to HMOD, providing new insights into the biomarker’s role in HT complications. Current evidence suggests that SCUBE-1 may be involved in mechanism of HT and HMOD through particularly endothelial dysfunction, platelet activation and inflammation.

Hypertensive phenomena are associated with blood vessel damage across multiple systems. In particular, retinopathy is characterized by damage and changes to the retinal vessels due to high blood pressure [24]. The retina, with its specialized capillary bed of endothelial cells, is particularly sensitive to vascular changes and damage due to its direct exposure to blood vessels [25]. As a result, hypertensive retinopathy may show more pronounced vascular changes and damage. Similarly, given the complex structure and functionality of the kidneys, hypertensive nephropathy may affect SCUBE-1 expression and function through multiple mechanisms. Microvascular damage, altered GFR, and inflammatory responses are factors that can elevate SCUBE-1 levels, reflecting renal injury [26]. In specific organs, such as the retina and kidney, damage may be concentrated in particular regions, whereas HT usually causes homogeneous damage to the heart. Although the damage resulting from HT in different organs is caused by similar mechanisms, the unique structures of each organ lead to varying sensitivities and specificities. Despite these differences, SCUBE-1 has been found to be diagnostically valuable for all three organ systems.

### Limitations

This study does have notable limitations. First, the analysis was conducted in a single-center setting, which may limit the generalizability of the findings to a broader, more diverse population. Second, patients receiving antihypertensive therapy may exhibit different SCUBE-1 levels or patterns of organ damage compared to newly diagnosed individuals. Third, the potential effects of specific antihypertensive medications on SCUBE-1 expression or HMOD should be considered as these factors may confound the results. Finally, variations in treatment regimens, medication adherence, and treatment duration may affect SCUBE-1 levels and contribute to variability in HMOD results.

## 5. Conclusions

The findings from this study suggest that SCUBE-1 levels can be used as a potential marker in diagnosing HMOD. In particular, the SCUBE-1 test demonstrated high specificity in detecting hypertensive retinopathy and high sensitivity for identifying renal damage. These findings support the potential use of SCUBE-1 in the early diagnosis of organ damage associated with HT, thereby improving clinical applications. Therefore, SCUBE-1 levels could be considered for routine screening or as a supplementary tool in the early diagnosis of organ damage in patients with HT.

## Data Availability

The datasets generated and/or analyzed during the current study are available from the corresponding author on reasonable request. All materials used in the study are also available upon request.

## Study Association

This article is part of the thesis of Doctoral submitted by Betül Ayça Yamak, from Gazi University.
